# Patients’ Perceptions and Attitudes to the Use of Artificial Intelligence in Breast Cancer Diagnosis: A Narrative Review

**DOI:** 10.3390/life14040454

**Published:** 2024-03-29

**Authors:** Filippo Pesapane, Emilia Giambersio, Benedetta Capetti, Dario Monzani, Roberto Grasso, Luca Nicosia, Anna Rotili, Adriana Sorce, Lorenza Meneghetti, Serena Carriero, Sonia Santicchia, Gianpaolo Carrafiello, Gabriella Pravettoni, Enrico Cassano

**Affiliations:** 1 Breast Imaging Division, IEO European Institute of Oncology IRCCS, 20141 Milan, Italy; luca.nicosia@ieo.it (L.N.); anna.rotili@ieo.it (A.R.); lorenza.meneghetti@ieo.it (L.M.); enrico.cassano@ieo.it (E.C.); 2 Postgraduation School in Radiodiagnostics, Università degli Studi di Milano, 20122 Milan, Italy; emilia.giambersio@unimi.it (E.G.); adriana.sorce@unimi.it (A.S.); 3 Applied Research Division for Cognitive and Psychological Science, IEO European Institute of Oncology, IRCCS, 20141 Milan, Italy; benedetta.capetti@ieo.it (B.C.); dario.monzani@unipa.it (D.M.); roberto.grasso@ieo.it (R.G.); gabriella.pravettoni@ieo.it (G.P.); 4 Department of Psychology, Educational Science and Human Movement (SPPEFF), University of Palermo, 90133 Palermo, Italy; 5 Department of Oncology and Hemato-Oncology, University of Milan, 20122 Milan, Italy; gianpaolo.carrafiello@unimi.it; 6 Foundation IRCCS Cà Granda-Ospedale Maggiore Policlinico, 20122 Milan, Italy; serena.carriero@policlinico.mi.it (S.C.); sonia.santicchia@policlinico.mi.it (S.S.)

**Keywords:** artificial intelligence, breast cancer, screening, diagnosis, psychological burden, population, survey, policy, healthcare

## Abstract

Breast cancer remains the most prevalent cancer among women worldwide, necessitating advancements in diagnostic methods. The integration of artificial intelligence (AI) into mammography has shown promise in enhancing diagnostic accuracy. However, understanding patient perspectives, particularly considering the psychological impact of breast cancer diagnoses, is crucial. This narrative review synthesizes literature from 2000 to 2023 to examine breast cancer patients’ attitudes towards AI in breast imaging, focusing on trust, acceptance, and demographic influences on these views. Methodologically, we employed a systematic literature search across databases such as PubMed, Embase, Medline, and Scopus, selecting studies that provided insights into patients’ perceptions of AI in diagnostics. Our review included a sample of seven key studies after rigorous screening, reflecting varied patient trust and acceptance levels towards AI. Overall, we found a clear preference among patients for AI to augment rather than replace the diagnostic process, emphasizing the necessity of radiologists’ expertise in conjunction with AI to enhance decision-making accuracy. This paper highlights the importance of aligning AI implementation in clinical settings with patient needs and expectations, emphasizing the need for human interaction in healthcare. Our findings advocate for a model where AI augments the diagnostic process, underlining the necessity for educational efforts to mitigate concerns and enhance patient trust in AI-enhanced diagnostics.

## 1. Introduction

Breast cancer has long stood as one of the most prevalent forms of neoplastic diseases affecting women globally. Its prominence among the most common forms of cancer has persisted over decades, shaping healthcare strategies and research endeavors worldwide. The year 2020, in particular, marked a significant landmark, with an estimated 19.3 million new cancer cases reported and nearly 10 million cancer-related deaths recorded worldwide. Among these statistics, breast cancer emerged as the most common, with approximately 2.2 million new cases diagnosed and close to 685,000 deaths attributed to the disease, thus being more ordinarily diagnosed even than lung cancer [1]. Breast cancer in the male population represents a rare entity with an estimated incidence of 1.2 per 100,000 in the US. Specific risk factors such as gynecomastia, BRCA mutations, Klinefelter syndrome, previous radiation exposure to the chest, and high estrogen levels are tightly linked to these diagnoses; therefore, routine screening mammography is not recommended for asymptomatic men [2].

The evolution of artificial intelligence (AI) in medical imaging and diagnostics has ushered in an era of precision medicine, significantly impacting breast cancer detection and management. AI algorithms, particularly in mammography [3], have demonstrated potential in enhancing diagnostic accuracy, reducing false positives and negatives, aiding risk stratification and prognostication [4], and sensibly reducing the time to examine images, which are very useful in breast cancer screening [5,6].

However, the successful implementation of AI in clinical practice hinges not only on its technical efficacy but also on patients’ acceptance and attitudes towards this technology. Understanding patients’ perceptions is crucial in the context of breast cancer diagnosis, where the psychological burden of screening and diagnosis is substantial [7]. Patient attitudes towards AI in healthcare can influence their willingness to engage with AI-assisted diagnostic procedures and can impact their trust in the outcomes of such diagnostics.

Patients’ attitudes towards AI in medicine are a topic of growing interest. Attitudes of patients towards AI differ, as some are optimistic about its potential to enhance healthcare while others harbor concerns, especially about possible misdiagnosis and privacy breaches [8]. Additionally, research has shown that patients generally prefer human doctors to AI-powered machines in diagnosis, screening, and treatment [9]. Overall, these findings underscore the importance of understanding and addressing patient attitudes towards AI in medicine.

The primary aim of this narrative review is to elucidate patient perceptions regarding the potential role of deep-learning algorithms in the detection of breast cancer. Specifically, we sought to understand patient apprehensions about the use of AI software in routine radiological practice. Central to our analysis are questions about patient trust, such as whether patients show more confidence in the clinical judgment of radiologists compared to AI predictions, or if there is a noticeable shift towards relying on algorithmic analysis. Additionally, we aimed to determine the extent of AI involvement that patients find acceptable or preferable in their diagnostic journey, thus providing insights that could guide the integration of AI in medical practice in a manner that is sensitive to patient needs and concerns.

We performed this narrative literature review utilizing databases such as PubMed, Embase, Medline, and Scopus, spanning publications from January 2000 to December 2023. Our search strategy involved a carefully constructed string of key terms to ensure a thorough exploration of the relevant literature. The search string employed was: (“artificial intelligence” OR “AI”) AND (“breast cancer” OR “mammography”) AND (“patient perspective” OR “patient opinion” OR “quality of life” OR “QoL”) AND (“screening” OR “diagnosis” OR “radiology”).

Through this meticulous approach, our initial search yielded a total of 49 results. Subsequently, we subjected these findings to a double reading assessment of the entire papers, excluding 42 papers and selecting 7 studies that contribute to shed light on the intricate relationship between AI, breast cancer diagnosis, and the patient experience, allowing for a deeper exploration of this critical field [9,10,11,12,13,14,15].

## 2. Receiving a Diagnosis of Breast Cancer

### 2.1. The Physical and Psychological Aftermath

Receiving a breast cancer diagnosis marks the onset of a challenging journey, encompassing not only the physical battles against the disease but also confronting its psychological repercussions. [16] Breast cancer exhibits a notable frequency of coexisting conditions, including psychological discomfort [17,18,19] issues related to anxiety and mood [20,21], feelings of depression [22], and enduring fatigue coupled with reduced social engagements, emerging as prevalent reactions to the diagnosis and therapeutic interventions associated with breast cancer [23]. In particular, it has been observed that the psychological impact of the illness is significant, especially in the transition to motherhood for women of childbearing age [19]. For these women, fears and concerns associated with a cancer diagnosis are primarily linked to the disease and its potential effects on pregnancy and the child’s health [19]. Furthermore, individuals diagnosed with primary breast cancer remain susceptible to enduring psychological challenges over an extended duration [24,25], underscoring the substantial influence of this health condition on the overall well-being of affected individuals.

The improvements in early cancer detection and efficacy of innovative treatments developed in recent years have supported a prolonged lifespan of cancer patients, generating, however, the onset of long-term psychological and physical consequences and altered quality of life [26]. During treatment, in fact, whether involving minor or major procedures, patients may grapple with temporary or permanent alterations to their bodies, giving rise to significant psychological challenge [27,28,29,30]. The removal of breasts, the development of swollen arms due to lymphedema, chemotherapy-induced baldness, pharmacologically triggered menopause, heightened skin sensitivity from radiation, and the use of prosthetics can impact the self-perception, body image, sexual function, and overall emotional well-being of women with breast cancer [16,31,32].

Moreover, 90% of breast cancer survivors experience sequalae following treatments, including a decline in physical strength of their upper body, and chronic neuropathic pain or nonpainful sensations in the amputated breast following surgery [33]. Accordingly, the integration of supportive measures, tailored to address both the physical and emotional strains, is essential in fostering resilience and recovery.

### 2.2. Psychological Burden of Carrying a BRCA Genetic Mutation

The discovery of a BRCA mutation carries with it not just a heightened risk for breast cancer but also a profound psychological burden, stemming from the anticipation of cancer and its implications on familial and personal health [34]. BRCA1 and BRCA2 stand out as the predominant genes associated with this specific cancer type compared to others [35]. It is reported that 55–72% of women who inherit a harmful BRCA1 variant and 45–69% of women who inherit a harmful BRCA2 mutation will develop breast cancer by 70–80 years of age, while the chances of developing breast cancer among the general population at some point in their lives is about 13% [36].

Several factors contribute to the decision-making process regarding preventive strategies among BRCA carriers. Some factors are linked to information processing [37] while others are associated with psychosocial variables such as risk perception, cancer-related worry, levels of emotion dysregulation, family history, and having young children [38,39].

The potential psychological responses and related considerations when a mutation is detected in an individual could include elevated levels of distress, anxiety, and depression [40]. These psychological manifestations can be attributed to an increased risk of future illnesses and implications not only for the health of the tested individuals but also for their entire family [41]. In addition, increased psychological distress may be triggered when genetic testing is conducted during a woman’s fertile age, highlighting the necessity also for fertility counseling [42]. Addressing these needs comprehensively can alleviate the psychological impact and empower women carrying a genetic mutation to make informed choices about their health [43,44].

### 2.3. Artificial Intelligence and Breast Cancer: Patients’ Perspectives

The integration of AI in medical diagnostics, particularly in breast cancer detection, is a rapidly evolving field. This evolution has prompted a need to understand the patient’s perspective on AI’s role in their healthcare. To address this gap, our research focuses on analyzing existing literature that explores patient attitudes towards AI in breast cancer diagnosis.

The main take-home messages of our review are summarized in Table 1.

In 2022, Borondy Kitts A.B. [41] reported that patient engagement in radiology AI revolves around two key areas: data sharing for AI development and AI’s use in patient care. Patients generally support data sharing if it benefits others or research but have concerns about privacy risks and trust issues. In terms of AI in medical care, patients are open to AI assisting radiologists but lack trust in unsupervised AI. They worry about liability, loss of human connection, and bias in AI algorithms. Building trust in AI requires transparency, security, and privacy measures. According to the author, radiologists can prepare patients by implementing data-sharing agreements for algorithm development and having discussions about AI use in their care. This presents an opportunity for radiologists to maintain strong patient relationships as AI becomes more integrated into healthcare.

In 2021, Ongena et al. [39] published the results of a survey administered to women undergoing screening mammography in the Netherlands; specifically, they investigated four precise themes regarding AI in radiology: trust and accountability (trust in AI in taking over diagnostic interpretation tasks of the radiologist, both with regard to accuracy, communication, and confidentiality), personal interaction (preference of personal interaction over AI-based communication), efficiency (belief in whether AI could improve diagnostic workflow), and a newly developed scale measuring the attitude towards AI in general medicine. They also took into consideration social status and the level of education of the patients. Their results showed that their population does not trust AI enough for its use in standalone interpretation of screening mammograms. Respondents were slightly more optimistic about the use of AI as a tool that could help select patients that require a second reader or not. However, a considerable proportion (41%) still opposed the idea of using AI as a tool to select patients for second reading. Seventeen percent of women explicitly objected against using AI as an actual second reader. Therefore, the combination of a radiologist as a first reader and an AI system as a second reader seems to be the most feasible approach to the population at present, as suggested also by the above-mentioned recent clinical trials [5,6].

In 2020, Lennox-Chhugani et al. [42] administered a similar survey that investigated topics concerning attitudes of women to the use of AI in the breast screening process in four National Health Service trusts providing acute care in the East Midlands of England. The study revealed that women generally have a limited understanding of the current mammogram reading process, with only a minority recognizing the involvement of two human readers in blind readings. However, sentiment analysis of free-text responses indicated that a significant proportion of women expressed positivity towards the use of AI in breast screening, with the largest percentage holding positive views. Additionally, thematic analysis highlighted perceived benefits of AI in breast screening, including increased efficiency, improved reliability, and greater safety. Many women expressed the belief that AI integration in breast screening is inevitable and beneficial for the future. Interestingly, women of screening age showed a higher inclination towards positive views on AI in breast screening compared to younger women, despite being less likely to use AI in everyday health advice or hold positive views of its impact in society.

In 2020, Adams et al. [43] investigated similar topics by a roundtable discussion where radiologists engaged with patients and invited them to share their opinions and concerns about the use of AI in radiology. They noted that the four themes that recurred the most during their conversation were the following: fear of the unknown, trust, human connection, and cultural acceptability. On the other hand, patients agreed that AI could have a positive impact on the workflow of radiologists by improving access and reducing waiting times, reducing time to diagnosis, and even increasing diagnostic accuracy.

In 2021, Pesapane et al. [9] conducted studies, including a survey, to investigate the attitudes and perceptions of patients towards the use of AI in mammography. Researchers administered an anonymous questionnaire to participants in a breast cancer screening program, focusing on their opinions about the introduction of AI in mammography. This questionnaire was developed in collaboration with psycho-oncologists and subsequently validated. The findings revealed that a significant portion of the sample (88%) held a positive view of AI’s role in mammography screening, recognizing its potential utility and security. Notably, 94% of respondents believed that radiologists should always provide their interpretation of mammograms. Furthermore, 90% opined that AI could assist in identifying cases warranting further investigation. A substantial majority (77%) concurred that AI should be employed at least as a secondary reader. A critical insight emerged regarding the attribution of responsibility for potential AI errors. About 52% of the participants believed that both software developers and radiologists share the responsibility for any mistakes made by AI systems. The survey also uncovered intriguing variations in opinions across different demographics. Women from diverse age groups and educational backgrounds exhibited distinct perspectives on AI’s potential use and involvement in medicine, highlighting the importance of considering demographic factors when assessing patient attitudes towards AI in healthcare. Women with a higher education level (e.g., high school diploma or university degree) were positively associated with optimistic thinking on the use of AI, although some concern was also observed among the more educated. Particularly, authors reported a lower perceived accuracy in medical AI knowledge as educational level increased. This subjective evaluation of personal knowledge about medical AI was explained by the “Dunning–Kruger” [45] effect, which describes how people with limited skills or knowledge in an area of expertise tend to overestimate their own knowledge or competence in that domain. Also, according to this survey, women held both software and radiologists accountable for errors.

The matter of accountability of errors when implementing AI is extremely controversial. Standardized AI governance frameworks and proper AI regulation and legislation are still loosely defined and largely underdeveloped [46]. The attribution of responsibility by patients is related to their understanding of the AI apparatus and workflow, which is not always fully explainable due to the “black box” nature of its networks [47,48]. The concept of “explainable AI” has recently been developed to unravel the inexplicable algorithms of AI in order to address and resolve ethical and legal issues, also concerning fault and accountability, and to make its use in clinical practice more acceptable and understandable by patients [49].

Additionally, in 2022, Bunnel and Rowe [44] investigated the effects that the implementation of AI in breast imaging could have on the relationship between the radiologist and the patient and their ways of communication. The results of their analysis showed that patients perceive and appreciate the competency of the radiologist by mutual effective communication and human interpretation of AI-generated diagnoses. According to patients, radiologists are able to administer adequate care when their competency and expertise are unaffected by AI integration, and they effectively identify potential AI errors.

The key findings of these investigations and surveys are summarized in Table 2.

Interestingly, a survey concerning similar topics was conducted in the UK by de Vries et al. [50] that, on the other hand, was aimed at the evaluation of the opinions of screening readers on the use and future applications of AI in mammography. Accredited breast cancer screening readers were asked to respond and give their opinions on four different scenarios for future, possible utilization of AI: a “partial replacement scenario” with a specialist and an AI algorithm examining the mammograms where, in case of disagreement, a different specialist would make the final decision; a “total replacement scenario” with AI algorithms examining the mammograms without input from radiologists, thus making the final decision; a “triage scenario” with AI algorithms initially examining the mammograms where, if suspicious findings are detected, a specialist would be required to review the image; a “companion scenario” with mammograms continuing to be examined by specialists as is the current practice, with on-demand access to an AI algorithm to help them make their decisions.

The data obtained from the survey evidenced that breast screening readers in the UK favor the introduction of AI, with over 63% of participants having a positive or strongly positive view of AI use in screening. Respondents overall preferred partial replacement (AI replaces one human reader) over other AI implementation scenarios. They objected to the total replacement scenario, while views on the triage and companion scenarios were mixed.

Some comments added by the responding radiologists also suggested other possible uses of AI in the screening setting, such as maximizing image quality, interpreting breast density, and then assessing risk and possible masking from breast density and fat—the parenchyma ratio—so that the algorithm can suggest whether or not to perform tomosynthesis. Approximately half of the respondents thought first readers (52%) and second readers (51%) should have access to the AI opinion. Most respondents (68%) thought that third readers or an arbitration panel should have access to the AI opinion.

In summary, the collective findings extracted from the referenced articles and surveys conducted among patients and breast radiologists alike score a harmonious consensus. Both groups emphasize the insufficiency of exclusively relying on AI for mammogram assessment, preferring instead its partial integration into the decision-making process. AI should function as an auxiliary tool, potentially assuming the role of a second or third reader in conjunction with a human radiologist. Such a collaborative approach not only optimizes diagnostic accuracy but also values the continued significance of human expertise and judgment in breast cancer detection and diagnosis.

Finally, we must acknowledge that the studies considered in this review predominantly originate from populations residing in medium- to high-income countries. These countries are typically at the forefront of adopting and integrating AI applications into various domains, including healthcare. Moreover, their populations tend to be more aware of technological advancements and medical innovations [9]. However, it is crucial to acknowledge that the impact of AI in healthcare, particularly in fields such as breast cancer screening, is not limited to affluent nations, as the implementation of AI holds significant promise in addressing healthcare disparities, especially in low-income countries [51,52,53]. As low-income countries often face challenges in establishing and maintaining comprehensive screening programs due to limited resources, infrastructure, and healthcare accessibility [54], AI has the potential to be a transformative tool, offering more accessible and cost-effective solutions for breast cancer screening [53]. It could, in fact, help provide easy access to better healthcare, such as screening mammography for vulnerable and at-risk women through algorithm-assisted, telemedicine-based platforms [55].

However, a paradox emerges when considering the global application of AI in healthcare. While AI has the potential to reduce healthcare disparities by improving access to screening and diagnostic services in low-income countries, the current concentration of AI-related research and development in high-income regions poses a challenge. The majority of AI algorithms are developed and validated on datasets primarily derived from affluent populations, which may not adequately represent the diversity of health conditions, demographics, and healthcare systems in low-income countries [7].

Furthermore, the adoption of AI in low-income regions can be hindered by several factors, including limited access to high-quality medical data for algorithm training, inadequate infrastructure, and a lack of awareness and acceptance among healthcare providers and communities. These challenges may inadvertently exacerbate healthcare disparities, as the benefits of AI may not be equally accessible to all populations [52].

To address these issues, initiatives should focus on developing AI solutions that are adaptable to resource-constrained settings, promoting data sharing and collaboration, and fostering education and awareness about the potential benefits of AI in healthcare. Efforts to expand the reach of AI in healthcare should be guided by a commitment to inclusivity, equitable access, and a thorough understanding of the specific challenges faced by underserved populations [56]. Only then can AI fulfill its promise of reducing disparities rather than accentuating them.

## 3. Conclusions

While recognizing AI’s potential for enhanced diagnostic accuracy and efficiency in mammography, patients express varied concerns about trust, personal interaction, and accountability, highlighting the need for a balanced approach in clinical practice.

Demographic differences in perceptions and concerns underline the importance of tailored patient education about medical AI. Legal and ethical considerations, particularly regarding error accountability and AI’s “black box” nature, necessitate the resolute development of an ever-increasing explainable AI [57] as well as standardized ethics and governance frameworks [58] capable of ensuring the ethical sustainability of AI and of maintaining and strengthening patient trust [59].

Nevertheless, a fundamental ethical requirement, reported by participants themselves, remains that of considering AI always as an empowering and enabling tool, which should never replace human evaluation of images altogether or hinder the direct interaction and communication between the radiologist and the patient.

In conclusion, the integration of AI presents substantial advancements in breast cancer screening. However, its effective clinical implementation necessitates addressing patient concerns and preserving the crucial role of radiologists in providing empathetic patient care. Moving forward, it is essential to prioritize efforts aimed at aligning AI technology with patient preferences and requirements, ensuring that AI complements rather than replaces the human aspects of healthcare delivery.

## Figures and Tables

**Table 1:** A concise overview of the major findings and implications from our review.

Aspect	Take-Home Messages
AI’s Potential in Diagnosis	AI enhances diagnostic accuracy and efficiency in breast cancer screening.
Patient Concerns	Varied concerns about AI’s trustworthiness, personal interaction, and accountability.
Role of Radiologists	Patients prefer AI as a complement to radiologists, not a replacement.
Demographic Variations	Perceptions of AI vary by demographic; tailored patient education is crucial.
Legal and Ethical Considerations	Need for explainable AI and governance frameworks to address legal/ethical issues.
Future Focus	Harmonize AI with patient needs, ensuring it supports human elements of healthcare.

**Table 2:** A synopsis of the key findings in the research and surveys conducted by the authors referenced in our review.

Study	Key Findings
Borondy Kitts (2022) [41]	- Patients support data sharing for AI development but have concerns about privacy and trust. - They are open to AI assisting radiologists but lack trust in unsupervised AI. - Building trust in AI requires transparency and privacy measures.
Ongena et al. (2021) [39]	- Population lacks trust in AI for standalone interpretation of mammograms. - Slightly more optimistic about AI assisting in patient selection for further review. - Prefer the combination of a radiologist as the first reader and AI as the second reader
Lennox-Chhugani et al. (2020) [42]	- Women express positivity towards AI in breast screening, citing increased efficiency and reliability. - Many believe AI integration in breast screening is inevitable and beneficial for the future
Adams et al. (2020) [43]	- Patients express fear of the unknown and concerns about trust and human connection regarding AI in radiology. - They believe AI could positively impact radiologists’ workflow
Pesapane et al. (2021) [9]	- Majority of participants hold a positive view of AI’s role in mammography screening. - Most believe radiologists should always provide their interpretation of mammograms. - Patients hold both software developers and radiologists accountable for AI errors.
Bunnel and Rowe (2022) [44]	- Patients appreciate effective communication and human interpretation of AI-generated diagnoses by radiologists. - Radiologists are perceived as competent when their expertise is unaffected by AI integration.

## Data Availability

No new data were created or analyzed in this study. Data sharing is not applicable to this article.

## References

[1] Sung H., Ferlay J., Siegel R.L., Laversanne M., Soerjomataram I., Jemal A., Bray F., Bsc M.F.B., Me J.F., Soerjomataram M.I. (2021). Global Cancer Statistics 2020: GLOBOCAN Estimates of Incidence and Mortality Worldwide for 36 Cancers in 185 Countries. CA Cancer J. Clin..

[2] Khan N.A.J., Tirona M. (2021). An updated review of epidemiology, risk factors, and management of male breast cancer. Med. Oncol..

[3] Pesapane F., Trentin C., Ferrari F., Signorelli G., Tantrige P., Montesano M., Cicala C., Virgoli R., D’acquisto S., Nicosia L. (2023). Deep learning performance for detection and classification of microcalcifications on mammography. Eur. Radiol. Exp..

[4] Pesapane F., Battaglia O., Pellegrino G., Mangione E., Petitto S., Manna E.D.F., Cazzaniga L., Nicosia L., Lazzeroni M., Corso G. (2023). Advances in breast cancer risk modeling: Integrating clinics, imaging, pathology and artificial intelligence for personalized risk assessment. Future Oncol..

[5] Lång K., Josefsson V., Larsson A.-M., Larsson S., Högberg C., Sartor H., Hofvind S., Andersson I., Rosso A. (2023). Artificial intelligence-supported screen reading versus standard double reading in the Mammography Screening with Artificial Intelligence trial (MASAI): A clinical safety analysis of a randomised, controlled, non-inferiority, single-blinded, screening accuracy study. Lancet Oncol..

[6] Dembrower K., Crippa A., Colón E., Eklund M., Strand F. (2023). Artificial intelligence for breast cancer detection in screening mammography in Sweden: A prospective, population-based, paired-reader, non-inferiority study. Lancet Digit. Health.

[7] Pesapane F., Cassano E. (2023). Enhancing Breast Imaging Practices: Addressing False-Positive Findings, Personalization, and Equitable Access. Radiology.

[8] Khullar D., Casalino L.P., Qian Y., Lu Y., Krumholz H.M., Aneja S. (2022). Perspectives of Patients about Artificial Intelligence in Health Care. JAMA Netw. Open.

[9] Pesapane F., Rotili A., Valconi E., Agazzi G.M., Montesano M., Penco S., Nicosia L., Bozzini A., Meneghetti L., Latronico A. (2023). Women’s perceptions and attitudes to the use of AI in breast cancer screening: A survey in a cancer referral centre. Br. J. Radiol..

[10] Campbell-Enns H., Woodgate R. (2015). The psychosocial experiences of women with breast cancer across the lifespan: A systematic review protocol. JBI Database Syst. Rev. Implement. Rep..

[11] Arnaboldi P., Riva S., Crico C., Pravettoni G. (2017). A systematic literature review exploring the prevalence of post-traumatic stress disorder and the role played by stress and traumatic stress in breast cancer diagnosis and trajectory. Breast Cancer Targets Ther..

[12] Arnaboldi P., Lucchiari C., Santoro L., Sangalli C., Luini A., Pravettoni G. (2014). PTSD symptoms as a consequence of breast cancer diagnosis: Clinical implications. SpringerPlus.

[13] Faccio F., Mascheroni E., Ionio C., Pravettoni G., Peccatori F.A., Pisoni C., Cassani C., Zambelli S., Zilioli A., Nastasi G. (2020). Motherhood during or after breast cancer diagnosis: A qualitative study. Eur. J. Cancer Care.

[14] Gallagher J., Parle M., Cairns D. (2002). Appraisal and psychological distress six months after diagnosis of breast cancer. Br. J. Health Psychol..

[15] Kissane D.W., Ildn J., Bloch S., Vitetta L., Clarke D.M., Smith G.C., McKenzie D.P. (1998). Psychological morbidity and quality of life in Australian women with early-stage breast cancer: A cross-sectional survey. Med. J. Aust..

[16] Fann J.R., Thomas-Rich A.M., Katon W.J., Cowley D., Pepping M., McGregor B.A., Gralow J. (2008). Major depression after breast cancer: A review of epidemiology and treatment. Gen. Hosp. Psychiatry.

[17] Denieffe S., Gooney M. (2011). A meta-synthesis of women’s symptoms experience and breast cancer. Eur. J. Cancer Care.

[18] Cohee A.A., Adams R.N., Fife B.L., Von Ah D.M., Monahan P.O., Zoppi K.A., Cella D., Champion V.L. (2017). Relationship Between Depressive Symptoms and Social Cognitive Processing in Partners of Long-Term Breast Cancer Survivors. Oncol. Nurs. Forum.

[19] Schmidt M.E., Wiskemann J., Steindorf K. (2018). Quality of life, problems, and needs of disease-free breast cancer survivors 5 years after diagnosis. Qual. Life Res..

[20] Dinapoli L., Colloca G., Di Capua B., Valentini V. (2021). Psychological Aspects to Consider in Breast Cancer Diagnosis and Treatment. Curr. Oncol. Rep..

[21] Izci F., Ilgun A.S., Findikli E., Ozmen V. (2016). Psychiatric Symptoms and Psychosocial Problems in Patients with Breast Cancer. J. Breast Health.

[22] Sebri V., Mazzoni D., Triberti S., Pravettoni G. (2021). The Impact of Unsupportive Social Support on the Injured Self in Breast Cancer Patients. Front. Psychol..

[23] Durosini I., Triberti S., Savioni L., Sebri V., Pravettoni G. (2022). The Role of Emotion-Related Abilities in the Quality of Life of Breast Cancer Survivors: A Systematic Review. Int. J. Environ. Res. Public Health.

[24] Sebri V., Durosini I., Mazzoni D., Pravettoni G. (2022). The Body after Cancer: A Qualitative Study on Breast Cancer Survivors’ Body Representation. Int. J. Environ. Res. Public Health.

[25] Lopes J.V., Bergerot C.D., Barbosa L.R., Calux N.M.d.C.T., Elias S., Ashing K.T., Domenico E.B.L.D. (2018). Impact of breast cancer and quality of life of women survivors. Rev. Bras. Enferm..

[26] Jing L., Zhang C., Li W., Jin F., Wang A. (2019). Incidence and severity of sexual dysfunction among women with breast cancer: A meta-analysis based on female sexual function index. Support. Care Cancer.

[27] Lovelace D.L., McDaniel L.R., Golden D. (2019). Long-Term Effects of Breast Cancer Surgery, Treatment, and Survivor Care. J. Midwifery Womens Health.

[28] Bjørnslett M., Dahl A.A., Sørebø Ø., Dørum A. (2015). Psychological distress related to BRCA testing in ovarian cancer patients. Fam. Cancer.

[29] Borreani C., Manoukian S., Bianchi E., Brunelli C., Peissel B., Caruso A., Morasso G., Pierotti M. (2014). The psychological impact of breast and ovarian cancer preventive options in *BRCA1* and *BRCA2* mutation carriers. Clin. Genet..

[30] Kuchenbaecker K.B., Hopper J.L., Barnes D.R., Phillips K.A., Mooij T.M., Roos-Blom M.J., Jervis S., Van Leeuwen F.E., Milne R.L., Andrieu N. (2017). Risks of Breast, Ovarian, and Contralateral Breast Cancer for BRCA1 and BRCA2 Mutation Carriers. JAMA.

[31] Landsbergen K.M., Brunner H.G., Manders P., Hoogerbrugge N., Prins J.B. (2010). Educational-support groups for BRCA mutation carriers satisfy need for information but do not affect emotional distress. Genet. Couns..

[32] Jamal L., Schupmann W., Berkman B.E. (2020). An ethical framework for genetic counseling in the genomic era. J. Genet. Couns..

[33] van Driel C.M.G., Oosterwijk J.C., Meijers-Heijboer E.J., van Asperen C.J., Zeijlmans van Emmichoven I.A., de Vries J., Mourits M., Henneman L., Timmermans D., de Bock G. (2016). Psychological factors associated with the intention to choose for risk-reducing mastectomy in family cancer clinic attendees. Breast.

[34] Guarino A., Polini C., Forte G., Favieri F., Boncompagni I., Casagrande M. (2020). The Effectiveness of Psychological Treatments in Women with Breast Cancer: A Systematic Review and Meta-Analysis. J. Clin. Med..

[35] Meiser B. (2005). Psychological impact of genetic testing for cancer susceptibility: An update of the literature. Psycho-Oncology.

[36] Knabben L., Siegenthaler F., Imboden S., Mueller M.D. (2022). Fertility in BRCA mutation carriers: Counseling BRCA-mutated patients on reproductive issues. Horm. Mol. Biol. Clin. Investig..

[37] Dibble K.E., Donorfio L.K.M., Britner P.A., Bellizzi K.M. (2022). Stress, anxiety, and health-related quality of life in BRCA1/2-positive women with and without cancer: A comparison of four US female samples. Gynecol. Oncol. Rep..

[38] Scotto L., Pizzoli S.F.M., Marzorati C., Mazzocco K., Pravettoni G. (2024). The impact of prophylactic mastectomy on sexual well-being: A systematic review. Sex. Med. Rev..

[39] Ongena Y.P., Yakar D., Haan M., Kwee T.C. (2021). Artificial Intelligence in Screening Mammography: A Population Survey of Women’s Preferences. J. Am. Coll. Radiol..

[40] Derevianko A., Pizzoli S.F.M., Pesapane F., Rotili A., Monzani D., Grasso R., Cassano E., Pravettoni G. (2023). The Use of Artificial Intelligence (AI) in the Radiology Field: What Is the State of Doctor–Patient Communication in Cancer Diagnosis?. Cancers.

[41] Borondy Kitts A. (2023). Patient Perspectives on Artificial Intelligence in Radiology. J. Am. Coll. Radiol..

[42] Lennox-Chhugani N., Chen Y., Pearson V., Trzcinski B., James J. (2021). Women’s attitudes to the use of AI image readers: A case study from a national breast screening programme. BMJ Health Care Inform..

[43] Adams S.J., Tang R., Babyn P. (2020). Patient Perspectives and Priorities Regarding Artificial Intelligence in Radiology: Opportunities for Patient-Centered Radiology. J. Am. Coll. Radiol..

[44] Bunnell A., Rowe S. (2023). The Effect of AI-Enhanced Breast Imaging on the Caring Radiologist-Patient Relationship. Pac. Symp. Biocomput..

[45] Kruger J., Dunning D. (1999). Unskilled and unaware of it: How difficulties in recognizing one’s own incompetence lead to inflated self-assessments. J. Pers. Soc. Psychol..

[46] Stogiannos N., Malik R., Kumar A., Barnes A., Pogose M., Harvey H., McEntee M.F., Malamateniou C. (2023). Black box no more: A scoping review of AI governance frameworks to guide procurement and adoption of AI in medical imaging and radiotherapy in the UK. Br. J. Radiol..

[47] Jeyaraman M., Balaji S., Jeyaraman N., Yadav S. (2023). Unraveling the Ethical Enigma: Artificial Intelligence in Healthcare. Cureus.

[48] Hulsen T. (2023). Explainable Artificial Intelligence (XAI): Concepts and Challenges in Healthcare. AI.

[49] Ali S., Abuhmed T., El-Sappagh S., Muhammad K., Alonso-Moral J.M., Confalonieri R., Guidotti R., Del Ser J., Díaz-Rodríguez N., Herrera F. (2023). Explainable Artificial Intelligence (XAI): What we know and what is left to attain Trustworthy Artificial Intelligence. Inf. Fusion.

[50] de Vries C.F., Colosimo S.J., Boyle M., Lip G., Anderson L.A., Staff R.T., Harrison D., Black C., Murray A., Wilde K. (2022). AI in breast screening mammography: Breast screening readers’ perspectives. Insights Imaging.

[51] Handtke O., Schilgen B., Mösko M. (2019). Culturally competent healthcare—A scoping review of strategies implemented in healthcare organizations and a model of culturally competent healthcare provision. PLoS ONE.

[52] Perry H., Eisenberg R.L., Swedeen S.T., Snell A.M., Siewert B., Kruskal J.B. (2018). Improving Imaging Care for Diverse, Marginalized, and Vulnerable Patient Populations. RadioGraphics.

[53] Pesapane F., Tantrige P., Rotili A., Nicosia L., Penco S., Bozzini A.C., Raimondi S., Corso G., Grasso R., Pravettoni G. (2023). Disparities in Breast Cancer Diagnostics: How Radiologists Can Level the Inequalities. Cancers.

[54] López-Gómez M., Malmierca E., de Górgolas M., Casado E. (2013). Cancer in developing countries: The next most preventable pandemic. The global problem of cancer. Crit. Rev. Oncol. Hematol..

[55] Malhotra K., Wong B.N.X., Lee S., Franco H., Singh C., Cabrera Silva L.A., Iraqi H., Sinha A., Burger S., Breedt D.S. (2023). Role of Artificial Intelligence in Global Surgery: A Review of Opportunities and Challenges. Cureus.

[56] Patel M.M., Parikh J.R. (2022). Education of Radiologists in Healthcare Disparities. Clin. Imaging.

[57] Floridi L., Cowls J. (2022). A Unified Framework of Five Principles for AI in Society. Machine Learning and the City.

[58] Morley J., Machado C.C.V., Burr C., Cowls J., Joshi I., Taddeo M., Floridi L. (2020). The ethics of AI in health care: A mapping review. Soc. Sci. Med..

[59] Mökander J., Floridi L. (2021). Ethics-Based Auditing to Develop Trustworthy AI. Minds Mach..

